# Association of HIV infection with distribution and viral load of HPV types in Kenya: a survey with 820 female sex workers

**DOI:** 10.1186/1471-2334-10-18

**Published:** 2010-01-26

**Authors:** Stanley MF Luchters, Davy Vanden Broeck, Matthew F Chersich, Annalene Nel, Wim Delva, Kishor Mandaliya, Christophe E Depuydt, Patricia Claeys, John-Paul Bogers, Marleen Temmerman

**Affiliations:** 1 International Centre for Reproductive Health (ICRH), Mombasa, Kenya; 2 International Centre for Reproductive Health (ICRH), Ghent University, Belgium; 3 Reproductive Health and HIV Research Unit (RHRU), University of Witwatersrand, South Africa; 4 International Partnership for Microbicides (IPM), Paarl, South Africa; 5 Coast Provincial General Hospital (CPGH), Mombasa, Kenya; 6 Applied Molecular Biology Research (AMBIOR), University of Antwerp, Belgium; 7 Laboratory for clinical pathology (Labo Lokeren - Campus Riatol), Antwerp, Belgium; 8 South African Centre for Epidemiological Modelling and Analysis, Stellenbosch University, South Africa

## Abstract

**Background:**

Human papillomavirus (HPV) and HIV are each responsible for a considerable burden of disease. Interactions between these infections pose substantial public health challenges, especially where HIV prevalence is high and HPV vaccine coverage low.

**Methods:**

Between July 2005 and January 2006, a cross-sectional community-based survey in Mombasa, Kenya, enrolled female sex workers using snowball sampling. After interview and a gynaecological examination, blood and cervical cytology samples were taken. Quantitative real-time PCR detected HPV types and viral load measures. Prevalence of high-risk HPV was compared between HIV-infected and -uninfected women, and in women with abnormal cervical cytology, measured using conventional Pap smears.

**Results:**

Median age of the 820 participants was 28 years (inter-quartile range [IQR] = 24-36 years). One third of women were HIV infected (283/803; 35.2%) and these women were y more likely to have abnormal cervical cytology than HIV-negative women (27%, 73/269, versus 8%, 42/503; *P *< 0.001). Of HIV-infected women, 73.3% had high-risk HPV (200/273) and 35.5% had HPV 16 and/or 18 (97/273). Corresponding figures for HIV-negative women were 45.5% (229/503) and 15.7% (79/503). After adjusting for age, number of children and condom use, high-risk HPV was 3.6 fold more common in HIV-infected women (95%CI = 2.6-5.1). Prevalence of all 15 of the high-risk HPV types measured was higher among HIV-infected women, between 1.4 and 5.5 fold. Median total HPV viral load was 881 copies/cell in HIV-infected women (IQR = 33-12,110 copies/cell) and 48 copies/cell in HIV-uninfected women (IQR = 6-756 copies/cell; *P *< 0.001). HPV 16 and/or HPV 18 were identified in 42.7% of LSIL (32/75) and 42.3% of HSIL (11/26) lesions (*P *= 0.98). High-risk HPV types other than 16 and 18 were common in LSIL (74.7%; 56/75) and HSIL (84.6%; 22/26); even higher among HIV-infected women.

**Conclusions:**

HIV-infected sex workers had almost four-fold higher prevalence of high-risk HPV, raised viral load and more precancerous lesions. HPV 16 and HPV 18, preventable with current vaccines, were associated with cervical disease, though other high-risk types were commoner. HIV-infected sex workers likely contribute disproportionately to HPV transmission dynamics in the general population. Current efforts to prevent HIV and HPV are inadequate. New interventions are required and improved implementation of existing strategies.

## Background

Human papillomavirus (HPV) is the most common sexually transmitted infection (STI) worldwide. It is the pre-eminent etiologic agent of cervical cancer, the second most common cancer among women [1]. Over 100 HPV types have been identified, about 20 of which are termed high-risk types, because of their propensity to disrupt control of normal cell-cycles and thereby foment the development of cervical malignancy [2]. In addition to cervical neoplasia, HPV is also causally associated with anogenital, oropharyngeal and some skin cancers [3,4]. HIV, another STI with extensive public health impact [5], shares many common behavioural risk factors with HPV and the infections interact in important ways. Female sex workers (FSW) have a particularly high risk for both HIV and HPV infection, and the interactions between these infections are especially pronounced in this population group [6-8].

Women with HIV infection commonly have a broader range of HPV genotypes, often with multiple concurrent HPV infections [9]. HIV disease influences the natural history of HPV by increasing the likelihood of persistent infection and virulence, and hastening the time-course of HPV disease [10]. Thus, compared with HIV-negative women, HIV-positive women are more likely to progress to cervical cancer, have a worse prognosis and a higher risk of recurrence [11-13]. It is also important to note that the interplay between HIV and HPV infection, and the consequences thereof are influenced by other factors such as age, sexual practices and socio-economic characteristics of women [14-16].

Besides the effects of HIV on the natural history of HPV infection, it is possible that HIV affects the transmissibility of HPV, and even possibly vice versa [17-19]. Causality, and the potential bidirectional nature thereof, cannot yet be definitively determined from available, mostly cross-sectional evidence. It remains plausible, however, that enhanced friability and the recruitment of CD4 and dendritic cells with HPV infection increases susceptibility to HIV acquisition in a manner similar to other genital tract infections such as *Neisseria gonorrhoea*.

The HPV vaccine, including high-risk HPV types 16 and 18, is effective in reducing HPV infection and HPV-related disease in women, and potentially in men [20]. These vaccines are increasingly becoming available in resource-constrained settings [21], which carry the overwhelming burden of HPV disease. Additional information about the prevalence of high-risk HPV types and their relative contribution to cervical disease in this setting would assist in planning for vaccine implementation and future development. This study assesses the prevalence of high-risk HPV types and HIV among FSW in Mombasa, Kenya, and the associations between these infections and with other demographic and behavioural variables. Additionally, the complex inter-relationships between type-specific HPV viral load, HIV infection and cervical cytology are evaluated.

## Methods

### Setting and population

Mombasa, in Kenya's Coast province, is a major economic centre in the region, with important tourism, port, rail and industrial enterprises, as well as a large FSW population. Sex workers in Mombasa are working either full- or part-time from bars, hotels, streets or from their homes [22,23]. About a third are involved in other small businesses, including selling foodstuffs, vegetables and, in some areas, local brew on the roadside [23]. Commonly the clients of sex workers are employed at local factories or are *matatu *(minibus taxi) touts or drivers.

### Study Design

Between July 2005 and January 2006, 820 FSW from Chaani and Kisauni divisions of Mombasa were enrolled in a community-based, cross-sectional survey. FSW were recruited using snowball sampling, a method appropriate for locating difficult-to-reach populations, particularly those with stigmatized behaviours or professions [24]. Full details of study methodology are provided elsewhere [23]. In brief, initial respondents were identified from bars, guest houses and the street, with subsequent participants recruited using snowball sampling. To limit the potential for friendship bias, we restricted the maximum number of women recruited through one participant to 10. Eligible participants were self-reported FSW, older than 16 years and working within Kisauni or Chaani. For this study, sex workers were defined as any woman who reported having received money or gifts in exchange for sex in the past year. The Kenyatta National Hospital Ethics and Review Committee reviewed and provided approval for the study procedures.

### Study procedures

In the two study areas, an existing drop-in centre for FSW and a municipality run health centre were modified to include clinical examination areas and related research facilities. Here staff obtained written informed consent, collected demographic and behavioural data using structured questionnaires, and offered HIV counselling and same-day testing. Blood plasma samples were taken and a gynaecological examination was done with speculum insertion, and collection of endocervical and high vaginal swabs.

Cervical samples were collected using a cervix brush (Cervex-brush^®^, Rovers^®^, Oss, The Netherlands) and cervical cytology was assessed with conventional Papanicolaou (Pap) smears. Slides were read by a cytologist with masters level training, supervised by a pathologist. An external cytopathologist provided quality control. The Bethesda Reporting System was used for cytologic classification [25]. The cervix brush tips were preserved in a liquid-based cytology collection medium (SurePath^®^, Tripath Imaging Inc., Burlington, North Carolina, USA) and stored at 4°C until further processing.

HPV testing was done as described by Depuydt et al [26] in an accredited laboratory (ISO certification: ISO15189). Briefly, HPV DNA was extracted from exfoliated cervical cells using the standard proteinase K-based digestion protocol, following the manufacturer's instructions. Cells were incubated with proteinase K solution (100 μg/ml) for 3 hours at 55°C. DNA was then further purified by spin column chromatography. HPV types were determined using a series of real-time PCR reactions with specific primers and TaqMan^® ^(Invitrogen, La Jolla, USA) probes for high-risk HPV types 16, 18, 31, 33, 35, 39, 45, 51, 52, 53, 56, 58, 59, 66 and 68 [2]. Low-risk HPV types 6 and 67 were also detected.

A parallel testing algorithm was used for HIV diagnosis using rapid immunoassays: Uni-Gold™ Recombigen^® ^HIV (Trinity Biotech plc, Bray, Ireland) and Determine^® ^HIV-1/2 (Abbott Japan co Ltd, Minato-Ku, Tokyo, Japan). In the case of indeterminate results, an enzyme-linked immunosorbent assay was used to confirm HIV status.

Where indicated, FSW received STI treatment free of charge. Women with a positive HIV test were referred to a nearby HIV clinic where medical care and antiretroviral treatment are provided, with no user fees. Women with abnormal cervical cytology were referred for further management and follow-up at the provincial teaching and referral hospital (Coast Provincial General Hospital, Mombasa) or a local municipal clinic supported by the International Centre for Reproductive Health.

### Data management and analysis

Data were double entered by separate data clerks. Following data checking and cleaning, analysis was done using Intercooled Stata, version 10.0 (Stata Corporation, College Station, TX, USA). The presence of any detectable viral load for specific HPV types was considered diagnostic for infection with that specific type. Chi-square tests were used to detect differences in HPV prevalence in women with and without HIV infection. For each HPV type, the Mann-Whitney *U *test was used to compare the HPV viral load of HIV infected and uninfected women who had ≥ 1 HPV copy/cell. Total HPV viral load was also calculated for each woman (the sum of the viral load of each high-risk HPV infection). To account for differences in age distribution between HIV infected and uninfected women, age-adjusted risk ratios are presented comparing the risk of HPV infection in these two groups. A multivariable logistic regression model was constructed, in which the presence of any high-risk HPV infection was the dependent variable. Fitting of the regression model was done using stepwise backward elimination, with variables retained if their removal markedly altered model parameters [27]. Variables associated with high-risk HPV in univariate analysis (*P *< 0.1) were included in the initial model. Associations between high-risk HPV infection and abnormal cervical cytology were also assessed.

## Results

### Socio-demographic and sexual behaviour characteristics

The median age of the 820 enrolled FSW was 28 years (IQR = 24-36 years; table 1). The vast majority were currently single, either never been married (346/818; 42.3%) or separated (33.0%, 270/818), divorced (13.0%, 106/818) or widowed (8.9%, 73/818). Women had a median of two children (IQR = 1-3 children).

**Table 1:** Distribution of demographics and sexual behaviour by HIV status and presence of high-risk HPV infection among female sex workers in Mombasa Kenya

		HIV-negative women			HIV-positive women		
					
Variable	All women	No high-risk HPV	High-risk HPV	*P*	No high-risk HPV	High-risk HPV	*P*
**Age **n/N (%)							
17-24	232/817 (28.4)	81/272 (29.8)	88/229 (38.4)		13/73 (17.8)	38/199 (19.1)	
25-29	216/817 (26.4)	72/272 (26.5)	64/229 (28.0)		14/73 (19.2)	54/199 (27.1)	
30-39	246/817 (30.1)	84/272 (30.9)	55/229 (24.0)		28/73 (38.4)	68/199 (34.2)	
≥40	123/817 (15.1)	35/272 (12.9)	22/229 (9.6)	0.02†	18/73 (24.7)	39/199 (19.6)	0.26†
median years (IQR)^¥^	28 (24-36)	28 (23-35)	27 (22-32)	0.02	32 (27-39)	30 (26-38)	0.27

**Religion **n/N (%)							
Catholic	255/820 (31.1)	76/274 (27.7)	67/229 (29.3)		26/73 (35.6)	77/200 (38.5)	
Muslim	236/820 (28.8)	89/274 (32.5)	78/229 (34.1)		12/73 (16.4)	42/200 (21.0)	
Protestant	313/820 (38.2)	101/274 (36.9)	78/229 (34.1)		34/73 (46.6)	80/200 (40.0)	
Other	16/820 (2.0)	8/274 (2.9)	6/229 (2.6)	0.92	1/73 (1.4)	1/200 (0.5)	0.62

**Education level **n/N (%)							
Never attended school	82/820 (10.0)	33/274 (12.0)	17/229 (7.4)		6/73 (8.2)	22/200 (11.0)	
Primary school	513/820 (62.6)	164/274 (59.9)	148/229 (64.6)		45/73 (61.6)	128/200 (64.0)	
Secondary school	202/817 (24.8)	67/274 (24.5)	57/229 (24.9)		20/73 (27.4)	49/200 (24.5)	
Tertiary education	22/817 (2.7)	10/274 (3.7)	7/229 (3.1)	0.35	2/73 (2.7)	1/200 (0.5)	0.38

**Marital status **n/N (%)							
Never married	346/818 (42.3)	113/274 (41.2)	109/228 (47.8)		25/73 (34.3)	78/199 (39.2)	
Married or cohabitating	23/818 (2.8)	15/274 (5.1)	4/228 (1.8)		0/0 (0)	5/199 (2.5)	
Separated, divorced or widowed	449/818 (54.9)	147/274 (53.7)	115/228 (50.4)	0.07	48/73 (65.8)	116/199 (58.3)	0.26

**Number of live children **n/N (%)							
0	114/819 (13.9)	39/274 (14.7)	29/229 (12.7)		16/73 (21.9)	18/199 (9.1)	
1	223/819 (27.2)	66/274 (25.1)	78/229 (34.1)		10/73 (13.7)	60/199 (30.2)	
2-3	331/819 (40.4)	119/274 (41.9)	85/229 (37.1)		29/73 (39.7)	82/199 (41.2)	
≥4	151/819 (18.4)	50/274 (18.4)	37/229 (16.2)	0.11	18/73 (24.7)	39/199 (19.6)	0.004

**Workplace **n/N (%)							
Bar or nightclub	580/820 (70.7)	179/274 (65.3)	167/229 (72.9)		52/73 (71.2)	149/200 (74.5)	
Home	163/820 (19.9)	63/274 (23.0)	44/229 (19.2)		14/73 (19.2)	33/200 (16.5)	
Street	54/820 (6.6)	23/274 (8.4)	10/229 (4.4)		4/73 (5.5)	16/200 (8.0)	
Hotel, guest house or other	23/820 (2.8)	9/274 (3.3)	8/229 (3.5)	0.17	3/73 (4.1)	2/200 (1.0)	0.31

**Age started sex work **mean years (sd) |	23.9 (6.8)	23.7 (6.4)	22.6 (6.7)	0.07	25.6 (7.0)	25.3 (7.2)	0.73

**Duration of sex work **median years (IQR)^¥^	4 (2 - 8)	4 (2-8)	4 (2-7)	0.51	6 (3-10)	5 (3-8)	0.24

**Number of partners **median in past week (IQR)^¥^	3 (2 - 6)	3 (2-6)	3 (2-6)	0.59	3 (2-5)	3 (1-6)	0.80

**Condom use at last sex with client **n/N (%)	661/819 (80.7)	210/273 (76.9)	195/229 (85.2)	0.02	58/73 (79.5)	161/200 (80.5)	0.85

**Condom use (clients) **n/N (%)							
Never	17/820 (2.1)	11/274 (4.0)	3/229 (1.3)		1/73 (1.4)	1/200 (0.5)	
Inconsistent	285/820 (34.8)	102/274 (37.2)	78/229 (34.1)		28/73 (38.4)	62/200 (31.0)	
Always	518/820 (63.2)	161/274 (58.8)	148/229 (64.6)	0.12	44/73 (60.3)	137/200 (68.5)	0.37

Chi^2 ^test unless indicated. ^¥ ^Mann-Whitney U test; † chi-square test for trend; | Student's *t *test^ Infection with HPV type 16, 18, 31, 33, 35, 39, 45, 51, 52, 53, 56, 58, 59, 66 and 68 defined as high-risk HPV infection

Women obtained clients mainly at bars or nightclubs (70.7%, 580/820), though about a fifth worked mainly from home (163/820). The median income from sex work per week was 14.3 US$ (IQR = 8.1-27.0 US$; exchange rate of US$1 = 74 Kenya shillings), obtained from a mean of 2.3 sex partners per week (standard deviation (sd) = 0.7). About two thirds of women reported consistently using a condom with clients (63.2%, 518/820), while consistent condom use with boyfriends was reported by only 92 of 437 women who currently had a boyfriend (21.1%).

### Prevalence of HIV

A third of participants were HIV infected (283/803; 35.2%); HIV status was unknown in 17 women. Compared to HIV-negative women, infected women were a median 3 years older. With each increase in decade of age, the odds of HIV infection increased 1.6 fold (95%CI = 1.4-2.0; *P *< 0.001). Prevalence of HIV was lower in Muslim (25.4%, 58/228) than in other women (39.1%, 225/575; *P *< 0.001) and women with a shorter duration of sex work (median 4 years [IQR = 2-7] for HIV-negative women versus median 5 years [IQR = 3-9] for positive women; *P *= 0.002). HIV prevalence was also higher in widowed women (62.9%, 44/70 compared with 32.6%, 238/731 in other women; *P *< 0.001) and in those with a recent genital sore (42.3%, 66/156 versus 33.6%, 217/646 in women without history of genital sores; *P *= 0.04).

### Prevalence of HPV infection

Of the 820 participants, study samples of adequate quality were obtained from 786 women for Pap smear assessment and from 789 women for HPV typing. More than half of FSW were infected with high-risk HPV (55.6%; 439/789). Among these women, over half had at least two different high-risk HPV types (54.9%; 241/439). HPV types 16 and/or 18 were detected in 22.8% of women (180/789), but more than twice as many were infected with high-risk HPV types other than 16 and 18 (48.5%, 383/789). A quarter of women were infected with low-risk HPV type 6 and/or 67 (194/789).

Co-infection with both HIV and high-risk HPV occurred in 25.8% (200/776) of FSW (table 2). High-risk HPV was detected in 73.3% (200/273) of HIV-infected women versus 45.5% (229/503) of uninfected women, with an age-adjusted risk ratio (RR) of 1.7 (95%CI RR = 1.5-1.9; *P *< 0.001). Co-infection with two or more high-risk HPV types was more frequent among HIV positive than negative women (48.7%, 133/273 versus 20.4%, 103/506; *P *< 0.001). Fifteen percent of HIV-infected FSW had four or more high-risk HPV types.

**Table 2:** Association of HIV status with cervical cytology and prevalence of high-risk HPV types in female sex workers in Mombasa Kenya

Variable	HIV-negative n/N (%)		HIV-positive n/N (%)		*P*	**Risk ratio**^**#**^ (95%CI)
**Prevalence of high-risk HPV**						
Any high-risk HPV infection*	N = 503	229 (45.5)	N = 273	200 (73.3)	<0.001	1.7 (1.5-1.9)
Type 16		56 (11.1)		66 (24.2)	<0.001	2.4 (1.7-3.4)
Type 18		34 (6.8)		51 (18.7)	<0.001	2.9 (1.9-4.6)
Type 31		31 (6.2)		33 (12.1)	0.004	2.0 (1.2-3.4)
Type 33		1 (0.2)		3 (1.1)	0.095	4.3 (0.41-45.8)
Type 35		39 (7.8)		49 (18.0)	<0.001	2.7 (1.7-4.1)
Type 39		20 (4.0)		36 (13.2)	<0.001	4.2 (2.3-7.5)
Type 45		18 (3.6)		21 (7.7)	0.012	2.3 (1.3-4.2)
Type 51		32 (6.4)		39 (14.3)	<0.001	2.5 (1.5-4.1)
Type 52		38 (7.6)		70 (25.6)	<0.001	3.7 (2.5-5.6)
Type 53		35 (7.0)		56 (20.5)	<0.001	3.0 (2.0-4.6)
Type 56		24 (4.8)		39 (14.3)	<0.001	2.9 (1.7-4.9)
Type 58		21 (4.2)		15 (5.5)	0.40	1.4 (0.7-2.7)
Type 59		43 (8.6)		36 (13.2)	0.04	1.7 (1.1-2.6)
Type 66		37 (7.4)		40 (14.7)	0.001	2.1 (1.3-3.3)
Type 68		2 (0.4)		6 (2.2)	0.02	5.5 (1.0-29.0)
Mean high-risk HPV types (sd)		0.8 (1.1)		1.8 (1.8)	<0.001	

**Low risk HPV**						
Type 6	N = 503	13 (2.6)	N = 273	10 (3.7)	0.40	1.4 (0.63-3.3)
Type 67		87 (17.3)		84 (30.8)	<0.001	1.8 (1.4-2.4)

**Viral load of high-risk HPV types (n≥1 copy/cell; median (IQR))**						
Type 16		38; 32.5 (4-472)		52; 145 (12-3278)	0.016	
Type 18		18; 12 (4-58)		31; 31 (6-718)	0.23	
Type 31		22; 15 (3-572)		25; 208 (58-13 257)	0.02	
Type 33		1; 204 (NA)		3; 15 453 (251-45 601)	0.18	
Type 35		34; 118 (14-4882)		48; 302 (61-9969)	0.11	
Type 39		14; 13 (1-89)		25; 106 (3-2600)	0.06	
Type 45		14; 8 (2-24)		21; 49 (4-252)	0.04	
Type 51		30; 43 (3-1738)		37; 826 (34-43 983)	0.02	
Type 52		31; 12 (3-71)		59; 76 (5-938)	0.05	
Type 53		27; 5 (2-14)		47; 31 (1-288)	0.14	
Type 56		20; 112 (5.5-829)		31; 219 (20-1511)	0.39	
Type 58		12; 16 (4.5-67)		13; 7 (4-32)	0.81	
Type 59		21; 27 (3-235)		20; 437 (20-3552)	0.04	
Type 66		22; 4 (2-794)		35; 48 (7-1193)	0.07	
Type 68		0; 0		1; 1; (NA)	-	
Total high-risk HPV viral load		48 (6-756)		811 (33-12 110)	<0.001	

**Cervical cytology (Pap smear)**						
Normal	N = 503	461 (91.7)	N = 269	196 (72.9)		
ASC-US/ASC-H		8 (1.6)		6 (2.2)		
LSIL		21 (4.2)		54 (20.1)		
HSIL		12 (2.4)		13 (4.8)		
Invasive carcinoma		1 (0.2)		0 (0)	<0.001	

^Infection with HPV type 16, 18, 31, 33, 35, 39, 45, 51, 52, 53, 56, 58, 59, 66 and 68 defined as high-risk HPV infection. *Any detectable HPV viral load >0 copies/cell considered diagnostic. #Age-adjusted risk ratio. LSIL low-grade intraepithelial lesion; HSIL high-grade squamous intra-epithelial lesion; ASC-US atypical squamous cells of undetermined significance; ASC-H atypical squamous cells: cannot exclude high-grade squamous intra-epithelial lesion. Median copies/cell are rounded to closest whole number. Vari. Cat. Variable category

### Predictors of high-risk HPV infection

Among the whole study population, age was not associated with prevalence of high-risk HPV. However, in HIV-negative women, age effects were noted. Here, HPV prevalence decreased with increasing age, for example, 52.1% of HIV-negative women aged 17-24 years had high-risk HPV (88/169) while this figure was 38.6% in women older than 40 years (22/57, *P *= 0.02; chi-square test for trend). Similarly, a linear decrease in the prevalence of HPV 16 and/or HPV 18 was noted in HIV-negative women, from a prevalence of 40.5% (32/169) in women below 25 years to 7.6% (6/57) in women above 40 years (odds of infection decreased by 0.71 with each increase in decade of age, 95%CI = 0.52-0.96; *P *= 0.03, chi-square test for trend). Among women with HIV infection, the prevalence of HPV 16 and/or HPV 18 did not vary across age groups.

When adjusted for age, having a child and condom use, HIV status remained a predictor for high-risk HPV infection (adjusted odds ratio [AOR] = 3.6; 95%CI = 2.6-5.1; *P *< 0.001; table 3). Having one or more children was also independently associated with high-risk HPV; these women had a 2.0 fold higher odds of high-risk HPV (95%CI AOR = 1.3-3.3; *P *= 0.003) than women with no children. High levels of reported condom use were not protective against high-risk HPV infection, nor were other measures of sexual behaviour or sex work duration associated with HPV prevalence.

**Table 3:** Association of HIV and other covariates with high-risk HPV infection: multivariable logistic regression analysis

Variable	Unadjusted odds ratio (95%CI)	Adjusted odds ratio (95%CI)	*P*
**Age**			
17-24	1.3 (0.80-2.0)	1.9 (1.1-3.2)	0.014
25-29	1.3 (0.83-2.1)	1.5 (0.92-2.5)	0.10
30-39	1.0 (0.66-1.7)	1.1 (0.69-1.7)	0.81
≥40	1.0	1.0	
**Have one or more children**			
No	1.0	1.0	
Yes	1.5 (1.0-2.3)	2.0 (1.3-3.3)	0.003
**Condom use with client at last sex**			
No	1.0	1.0	
Yes	1.5 (1.0-2.1)	1.4 (0.96-2.1)	0.08
**HIV infection**			
No	1.0	1.0	
Yes	3.3 (2.4-4.5)	3.6 (2.6-5.1)	<0.001

### Effect of HIV status on prevalence of specific high-risk HPV types

Type-specific analysis showed that the most prevalent HPV types were similar in HIV-positive and -negative women. In decreasing order, the most prevalent high-risk HPV types among HIV-positive women were 52, 16, 53, 18, 35 and 66, while for HIV-negative women the order was HPV 16, 59, 35, 52, 66 and 53 (table 2).

Compared with HIV uninfected women, prevalence of all HPV types was higher in HIV-infected women, with the age-adjusted risk ratio a median 2.7 fold higher (IQR RR = 2.1-3.7; range of RRs = 1.4-5.5). HPV 16 and/or HPV 18 infection was found in 35.5% (97/273) of HIV-positive women and in 15.7% (79/503) of HIV-negative women (*P *< 0.001).

### HPV viral load

HIV infection was significantly associated with an elevated HPV viral load for types 16, 31, 39, 45, 51, 52, 59 and 66. For 9 of the 15 high-risk HPV types measured, the median viral load for high-risk HPV was 5 or more times higher in HIV-positive than HIV-negative women. The total HPV viral load was 16.9 times higher in women with HIV infection compared to uninfected women (median 811 copies/cell versus 48 copies/cell; *P *< 0.001).

### Cervical cytology

High-risk HPV was strongly associated with abnormal cervical cytology, and was detected in 88.5% of women with HSIL (23/26) and 82.7% with LSIL (62/75). HPV type 16 and/or 18 was detected in 42.7% of women with LSIL (32/75) and 42.3% with HSIL (11/26). However, high-risk HPV types other than 16 and 18 were more frequent, occurring in 74.7% of LSIL (56/75) and 84.6% of HSIL (22/26), even more so among HIV-infected women (12 of 13 women with HSIL). High-risk HPV type 52 (907 copies/cell) and low-risk type 67 were detected in the woman who had invasive carcinoma on cytology.

Half of women with a normal Pap smear had high-risk HPV (327/648), though high-risk HPV was more common among HIV-infected women with normal cytology (67.4%; 130/193). Abnormal Pap smears were also markedly more common in HIV-infected women, of whom about 5% had HSIL (13/269) and 20% LSIL (54/269). The corresponding figures for HIV-negative women were 2.4% (12/503) and 4.2% (21/503), with one woman having squamous cell carcinoma. The 13 HIV-infected women with HSIL had a median of 3 high-risk HPV infections (IQR = 2-3).

Of the 51 HIV-infected women whose total HPV viral load was ≥10^4 ^copies/cell, 24 (47.1%) had LSIL and 6 had HSIL lesions (11.8%). Only 18 HIV-negative women had a total HPV viral load = 10^4 ^copies/cell, of whom 4 had LSIL and 1 HSIL.

## Discussion

In this study, about one-third of female sex workers were infected with HIV and more than half with high-risk HPV. In multivariable analysis, high-risk HPV was more than three times as common among HIV-positive than negative women. Prevalence of high-risk HPV in the study population is notably higher than in a meta-analysis of HIV-infected women including 20 studies, mostly from North America [28]. Though it is difficult to make direct comparisons between studies as HPV detection methods differ, the prevalence in this study is similar to a recent study among HIV-infected female hospital patients in Zambia [29]. Also of note, among HIV-infected women who had normal cervical cytology, two thirds had high-risk HPV infection in this study, twice as many as the meta-analysis of HIV-infected women [28]. Prevalence of high-risk HPV in HIV-negative FSW in our study is similar to previous reports among sex workers in other settings, for example, 39% in Spain [6], 43% in Mexico [30] and 48% in Japan [8], though a study in Calcutta, India reported higher levels (63%) [7].

Simultaneous infection with multiple HPV types was more frequent in HIV-positive women. This finding is consistent with previous reports [28], and according to Chaturvedi et al [31], is attributable to the common mode of transmission of HPV and HIV; the inability to clear HPV infections; as well as reactivation of latent HPV infections, the latter two occurring as a result of immune suppression [9,32-34]. Similar factors may underlie the concerning high levels of HPV 16 and 18 noted in older HIV-positive women in this study [35]. Though here, a particular ability of HPV 16 to evade immune surveillance in HIV-positive women may account for the high levels noted in all age groups [34].

Persistent HPV infection is the main cause of cervical cancer, and since this is more common in HIV-infected women, it makes the presence of cervical lesions in a quarter of HIV-positive women in this study even more concerning [36]. Many previous studies, including one in Mombasa [37], have noted a higher prevalence of HSIL among HIV-positive than HIV-negative women [38-40].

Broadly, the distribution of high-risk HPV types were similar in HIV-infected and uninfected women, albeit that the most common HPV types occurred in slightly differing order of prevalence. However, the most common HPV types in our study differ from those in other settings [28,40]. These regional differences in HPV types might, at least partly, be attributed to HIV and its differential effects on the natural history of specific types. However, not all findings support this assertion [41].

An association between parity and high-risk HPV has been noted previously, though might be accounted for by confounding due to sexual behaviour, such as unprotected sex or age at coital debut [6,35,42]. A large multicentre case-control study suggests, however, that full-term pregnancy is a cofactor that increases the risk of progression from HPV infection to cancer [35]. This, the authors speculate, might be due to hormonal changes in the second and third trimester of pregnancy or to increased cervical ectopy with multiparity. Possibly, the reduction in cervical cancer noted worldwide might be partly explained by the general global decline in parity.

Age, as previously shown [6,30,43,44], is an important predictor of HPV infection, with younger women being more frequently infected. Again, the absence of age effects among HIV-infected women in our study may be explained by a decreased ability to clear HPV infection, reactivation of HPV or re-infection with HPV types previously cleared, all of which likely occur more commonly in HIV-infected women.

Results presented here are consistent with a meta-analysis by De Vuyst et al. describing higher median viral loads of high-risk HPV in HIV-positive women, regardless of HPV type [45]. HPV viral load possibly correlates with the immune deficiency in these patients [10] and with likelihood of persistent HPV infection [46]. The highest viral burdens were found with HPV 33 in HIV-positive women and HPV 56 in HIV-negative women. HPV 16 had a relatively low viral load, though even at low viral levels has high oncogenic potential [47,48].

Even though current HPV vaccines offer some cross-protection against several HPV types [49], type-specific analysis of HPV data remains important for informing future HPV vaccine development and distribution. In women attending a family planning clinic in Nairobi, Kenya, less than 10% had HPV 16 and/or 18, and these types were present in about 40% of HSIL lesions [50]. In our study, HPV 16 and/or HPV 18 were present in about 20% of study participants, while still in about 40% of women with LSIL or HSIL. However, that most cervical lesions were negative for HPV 16 and/or 18, suggests that other high-risk HPV types might play a more important role in this context of female sex work. These types appear of particular importance in HSIL lesions in HIV-infected women [28]. It is also possible that the specific oncogenic HPV types that contribute to the development of cervical cancer in HIV-positive women may differ from those in HIV-negative women [51].

It is difficult to ascertain to what degree the sample obtained through snowballing methods is representative of the FSW population in Mombasa. Though snowball sampling is appropriate for locating difficult-to-reach populations, as it is a non-random sampling method, selection bias may occur. The investigators elected to have a large sample size, in an attempt to mitigate such effects. As a further limitation, information was missing on both HIV and high-risk HPV types, which could introduce bias. Such bias, however, is likely minimal as the missing information can be assumed to be non-differential in nature [52].

Patterns of HPV prevalence will likely alter the impact of prophylactic HPV vaccination [53]. The extent of this would be better understood if more information was available on the natural history of dysplasia associated with HPV types other than 16 and 18. The study population described here is highly suited to prospective assessment of this and related topics, given the considerable burden on HPV disease in this population. Moreover, previous cohorts in this study population have been feasible, with more than 90% cohort retention rates [54,55].

## Conclusion

Given the common occurrence of high-grade cervical lesions, especially in HIV-infected women, regular HPV screening and follow-up is essential for prevention of cervical cancer. Even though much is known about these diseases and their optimal control, current measures to prevent HIV and HPV in this population are clearly inadequate. The lack of control of these infections means that sex workers likely play a disproportionate role in transmission dynamics of both infections in the general population. Additional prevention measures, especially higher population-level coverage with HPV vaccine, are required.

## Competing interests

The authors declare that they have no competing interests.

## Authors' contributions

SL participated in the design of the study, supervised the study and contributed to interpretation of findings and drafting of the final manuscript. DVB and MFC assisted in drafting the manuscript. MFC and WD performed the data analysis. AN critically reviewed the manuscript and assisted in interpretation of the study findings. KM participated in the study coordination, collection of data and helped draft the manuscript. CED analysed the samples and assisted in interpretation of the results. PC, JPB and MT made a substantial contribution to the study design and interpretation of findings. All authors read and approved the final manuscript.

## Pre-publication history

The pre-publication history for this paper can be accessed here:

http://www.biomedcentral.com/1471-2334/10/18/prepub
